# Influence of human papillomavirus on semen parameters and male infertility: a single-center study

**DOI:** 10.5935/1518-0557.20250186

**Published:** 2026

**Authors:** Stefan Matik, Ana-Marija Bosilkovska, Simona Bardakoska-Stefanovska, Ivana Matik, Sotir Ropi, Aleksandar Jovanovski, Hristijan Trpchevski, Marija Petrusevska

**Affiliations:** 1 Private hospital for Obstetrics and Gynecology with IVF Center for Reproductive Medicine ‘Plodnost’, Bitola, North Macedonia; 2 Public clinical hospital ‘Dr. Trifun Panovski’, Bitola, North Macedonia; 3 Institute for preclinical and clinical pharmacology and toxicology, Medical faculty, University “Ss. Cyril and Methodius”, Skopje, North Macedonia

**Keywords:** HPV, semen analysis, male infertility, PCR

## Abstract

**Objective:**

To detect the presence of seminal HPV and examine its association with semen quality in men from infertile and fertile couples.

**Methods:**

This single-center case-control study involved 50 men from infertile couples and 10 proven-fertility men as controls. Semen samples collected were analyzed using computer-aided semen analysis and tested for HPV detection and genotyping with real-time PCR.

**Results:**

HPV DNA was detected in the semen of 9 men from infertile couples (18.0%) and 1 proven-fertility control (10.0%). Among infertile couples, HPV positivity was linked to significantly altered semen parameters: increased progressive motility (*p*=0.0399), higher presence of sperm with excess residual cytoplasm (*p*=0.05), and a lower percentage of normal acrosomes (*p*=0.05). Kinematic measurements, including straight-line velocity (VSL, *p*=0.0235), average path velocity (VAP, *p*=0.0434), linearity (LIN, *p*=0.05), wobble (WOB, *p*=0.05), and beat cross frequency (BCF, *p*=0.0239), were significantly higher in HPV-positive infertile men. Compared to fertile controls, HPV-positive men from the infertile group showed significantly lower sperm concentration (*p*=0.0493), total motile sperm count (*p*=0.0291), and mucus penetration ability (*p*=0.0088), along with a reduced percentage of morphologically normal sperm (*p*=0.05). Conversely, they had higher rates of sperm with excess residual cytoplasm (*p*=0.05), tail deformities (*p*=0.05), and neck and midpiece deformities (*p*=0.0499).

**Conclusions:**

Seminal HPV is associated with impaired sperm parameters in infertile men. This highlights HPV’s role as a potential contributing factor in male infertility, warranting further investigations.

## INTRODUCTION

Men with unexplained abnormal semen analysis results and those with normal test outcomes but unexplained infertility are becoming more common in clinical practice. Inflammatory processes and infections of the genital tract remain among the primary causes of male fertility problems (Akhigbe *et al*., 2022). Interest has increased in examining the effect of seminal human papillomavirus (HPV) infection on male infertility, semen quality, and reproductive outcomes. The lifetime likelihood of HPV infection in men reaches 90%, mainly due to the virus’s high contagiousness and the often asymptomatic nature of the infection (Bruni *et al*., 2023; Capra *et al*., 2022).

The HPV ontogenetic cycle has two phenotypic phases: infectious virions and the subsequent transient pathway of producing infectious virions in non-dividing cells (virions present on or in these cells-including spermatozoa and exfoliated epithelial cells in the ejaculate); and virus present in dividing cells, which is non-infectious and part of the non-infective, transformation-related cancer pathway. Regarding infertility and subfertility, the transient virion-producing infections are particularly relevant (Depuydt *et al*., 2016a; Depuydt *et al*., 2019; Gheit, 2019).

HPV affects spermatozoa by inhibiting Aquaporin 8, an important transmembrane channel responsible for transporting water, hydrogen peroxide, and small molecules that regulate the spermatozoon’s volume and oxidative stress (Pellavio *et al*., 2020). Infection with HPV does not eliminate the infected sperm’s fertilizing ability. In vitro studies show that HPV DNA localizes at the equatorial region of the sperm head through interaction of the viral capsid protein L1 with the syndecan-1 receptor (Foresta *et al*., 2011).

Numerous studies have linked the presence of HPV in semen with idiopathic asthenozoospermia, lower sperm concentration, abnormal morphology, idiopathic infertility, the presence of anti-sperm antibodies in the ejaculate, higher sperm DNA fragmentation, reduced viability, and longer time to spontaneous pregnancy. HPV-positive semen is associated with a lower intrauterine insemination success rate, increased risk of spontaneous abortion, and repeated miscarriages. Additionally, a decreased blastulation rate has been reported when using HPV-positive sperm for intracytoplasmic sperm injection. (Depuydt *et al.*, 2016a; Xiong *et al*., 2018; Wang *et al.*, 2021; Weinberg *et al.*, 2020; Moreno-Sepulveda & Rajmil, 2021; Siristatidis *et al*., 2018; Capra *et al*., 2022; Depuydt *et al*., 2016b; Garolla *et al*., 2016; Das *et al*., 2023). The role of HPV in medically assisted reproduction has been emphasized in a recent guideline (ESHRE Guideline Group on Viral infection/disease *et al*., 2021). However, current literature remains inconclusive, and data from clinical research and experimental studies are limited, warranting further investigation.

Nucleic acid amplification techniques (NAATs), especially polymerase chain reaction (PCR), are regarded as the gold standard for HPV detection and genotyping. Quantitative real-time PCR is commonly used for viral DNA measurement, broad target detection, and multiplexing (ESHRE Guideline Group on Viral infection/disease *et al*., 2021).

This study reports the results of a case-control investigation aiming to detect HPV DNA in the semen of men from both infertile and fertile couples. Additionally, we examined the relationship between the presence of viral DNA in semen and abnormal spermiogram parameters.

## MATERIAL AND METHODS

### Study design and population

**Figure 1:**
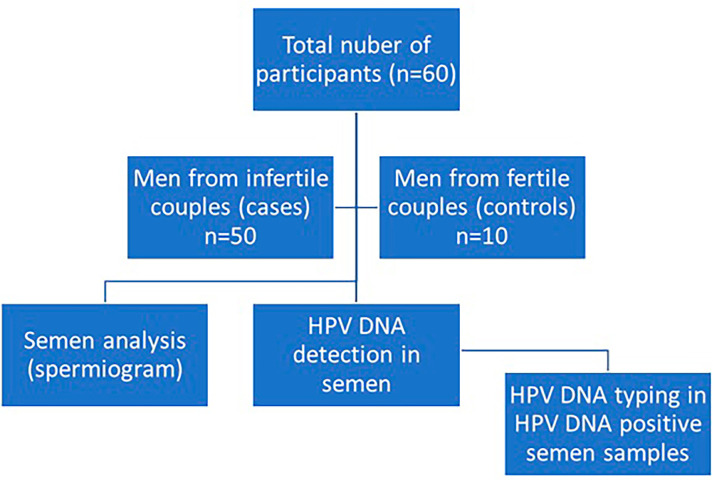
Study’s design flowchart.

This single-center case-control study included 60 men: 50 from infertile couples referred for semen analysis (infertile group) and ten with proven fertility as controls (fertile couples from the obstetrics department at the same institution). The study was conducted from June to November 2024. The flowchart of the study is shown in Figure 1.

Men who met the following criteria were included as men from infertile couples: previous attempts to achieve pregnancy with the same partner lasting at least one year; age: 18-50 years; partner’s age: 18-43 years; and no significant comorbidities in the female partner, such as cardiovascular diseases, metabolic syndrome, premature ovarian insufficiency, endometriosis with reduced ovarian reserve, anovulation with amenorrhea, hypogonadotropic hypogonadism, a history of cancer or cancer treatment, bilateral tubal obstruction, or bilateral salpingectomy.

Men who met the following criteria were included as fertile controls: age 18-50 years; proven fertility-either a clinical pregnancy at ≥22 weeks of gestation at the time or a live birth, both achieved after a maximum of one year of unprotected, regular sexual intercourse with the same partner.

All men with a history of cryptorchidism, testicular trauma, orchidectomy, mumps orchitis, signs or symptoms of genitourinary tract infection (such as cystitis, pyelonephritis, prostatitis, seminal vesiculitis, urethritis, or orchoepididymitis), professional exposure to radiation, extreme temperatures, pesticides, organic solvents, dyes, or varnishes, presence of clinical varicocele, previous treatment with hemotherapy, radiation therapy, medications with known reproductive toxicity, hypogonadotropic hypogonadism, azoospermia, an advanced partner’s age (>43 years old), or a previous pregnancy achieved via assisted reproduction were excluded from the study.

All participants were included after obtaining written informed consent in accordance with the protocol approved by two independent institutional Ethics Committees. The study was conducted in compliance with the ethical principles outlined in the Declaration of Helsinki (2013) and adhered to the STrengthening the Reporting of Observational Studies in Epidemiology (STROBE) statements for reporting observational trials (von Elm *et al*., 2008).

### Semen collection and analysis

Semen samples were obtained through masturbation after 2-7 days of sexual abstinence, following standard procedures for hand and genital hygiene. After liquefaction at room temperature, semen analysis was performed using 5 µL of ejaculate for wet preparation and a morphology stain (GoldyCyto SB^®^ pre-stained slides, Microptic S.L., Spain), with an automated computer-assisted semen analysis system (Microptic SCA, Spain) (World Health Organization, 2010). The analysis included macroscopic parameters such as volume, liquefaction, viscosity, and pH; microscopic parameters including sperm concentration, total sperm count, percentage of progressive and total (progressive plus nonprogressive) motility, total motile sperm count, and kinematic indices-VCL (curvilinear velocity), VSL (straight line velocity), VAP (average path velocity), LIN (linearity, VSL/VCL), STR (straightness, VSL/VAP), WOB (wobble, VAP/VCL), ALH (amplitude of lateral head displacement), BCF (beat cross frequency)-as well as the percentage of mucus penetration, morphologically normal spermatozoa, teratozoospermia (TZI), and sperm deformity (SDI) indices. Additionally, the analysis determined the percentage of abnormalities in the head, neck and midpiece, tail, excess residual cytoplasm, the proportion of sperm with normal and abnormal acrosomes, round cell concentration, and other relevant parameters.

### Multiplex real-time quantitative PCR for HPV DNA detection in semen

After liquefaction, part of the ejaculate was used for PCR. Briefly, 200 µL of ejaculate were processed with an automatic HPV DNA extraction system (SaMag-12^®^), following the manufacturer’s instructions. Sterile normal saline served as the negative control, and beta-globin gene as the internal positive control. Amplification, detection, and genotyping were performed using Quant-21^®^ (DNA Technology, Russia) - an in vitro diagnostic test for the specific identification and quantification of 21 HPV types (low-risk types: 6, 11, 44; potentially high-risk types: 26, 53, 66, 68, 73, 82; high-risk types: 16, 18, 31, 33, 35, 39, 45, 51, 52, 56, 58, 59).

### Statistical analysis

Data were described using numbers and/or percentages, or median and range, or mean and standard deviation (SD), or standard error of the mean (SEM), as appropriate. Differences between groups were explored using the t-test or Mann-Whitney test when the sample distribution was not normal. The Shapiro-Wilk test was performed to assess the normality of the data distribution. Due to unequal sample sizes (infertile group=50 and fertile group=10), tests robust to unequal variances, such as Welch’s t-test, were applied when needed. A priori sample size calculation was not performed because of the exploratory design and limited patient availability.

Since this is an interim analysis of an ongoing study, no formal sample size calculation was conducted at this point. Additionally, limited patient availability due to the research’s nature also contributed to the smaller fertile group. However, by applying the Holm-Sidak correction, the increased risk of Type 1 error inflation from multiple comparisons was controlled. This stepwise procedure offers more power than the Bonferroni correction while still controlling for Type 1 error.

A *p* value ≤0.05 was considered statistically significant. All statistical analyses were performed using Graph Prism 9 (USA).

## RESULTS

### Demographics and clinical characteristics of patients

Sixty men who met all inclusion criteria and did not meet any exclusion criteria were enrolled in the study. Nearly one-third (37.5%) of them were smokers. The average age ± SEM of the men included was 33.88±0.65 years for the infertile couples and 32.18±0.73 years for the fertile couples, with the difference not being statistically significant (*p*=0.1865, t-test). Both groups also showed no significant difference in the female partners’ mean age ± SEM (33±1.20 years for infertile couples *vs*. 30.9±1.96 years for fertile couples, *p*=0.0571, t-test).

Additionally, 14 of the participants (23.33%) used food supplements, with the most common being Ashwagandha, vitamin C, vitamin D, selenium, vitamin B6, taurine, and N-acetyl cysteine. Regarding the macroscopic parameters: semen volume (mean±SEM: 2.628±0.218 mL for infertile couples *vs*. 2.128±0.212 mL for fertile couples, *p*=0.3624, t-test) and pH value (7.53±0.012 for infertile couples *vs*. 7.539±0.028 for fertile couples, *p*=0.1156, t-test), as well as days of sexual abstinence before semen analysis (3.47±1.49 days in the infertile group and 2.81±1.18 days in the fertile group, *p*=0.0949, t-test), no statistically significant differences were found.

### Microscopic semen parameters in men from the infertile and fertile groups

The results from the microscopic semen analysis are shown in Table 1. Specifically, for VCL, VSL, and VAP, a statistically significant difference was observed between the infertile and fertile groups (*p*=0.0475, *p*=0.0378, *p*=0.0337; respectively, t-test). Additionally, a significant difference was found in the percentage of tail abnormality (*p*=0.0459, t-test), the percentage of morphologically normal spermatozoa (*p*=0.0498, t-test), BCF (*p*=0.0197, t-test), the percentage of head deformities (*p*=0.0097, t-test), and mucus penetration (*p*=0.0008, t-test). In the infertile group, ALH and the percentage of neck and midpiece abnormalities were higher and lower, respectively, with these differences being statistically significant (*p*=0.05 for ALH, and *p*=0.0491 for neck and midpiece abnormalities, t-test).

**Table 1:** Microscopic semen parameters, infertile vs. fertile couples^*^.

Parameter	Mean value ± SEM (infertile couples) n=50	Mean value ± SEM (fertile couples) n=10	*p*-value (t-test, Mann-Whitney)
**Sperm concentration (M/mL)**	30.80±5.43	53.25±14.8	0.0734
**Sperm count (M/sample)**	72.31±10.08	89.43±19.46	0.0989
**Progressive motility (%)**	39.19±3.39	48.07±5.037	0.1812
**Total motility (%)**	54.05±3.55	65.02±6.299	0.052
**Total motile sperm count (TMSC) (M/sample)**	34.60±6.11	56.81±16.93	0.1550
**VCL (µm/s)**	45.60±1.795	51.28±3.608	0.0479
**VSL (µm/s)**	21.34±0.8431	25.71±2.013	0.0398
**VAP (µm/s)**	29.30±1.015	34.17±1.988	0.0330
**LIN (%)**	47.55±1.682	49.12±1.966	0.4248
**STR (%)**	67.76±1.38	71.44±2.112	0.3479
**WOB (%)**	64.95±1.704	66.53±1.174	0.4606
**ALH (µm)**	2.159±0.088	2.456±0.146	0.0500
**BCF (Hz)**	6.278±0.1861	7.344±0.367	0.0197
**Mucus penetration (%)**	16.66±1.744	26.17±2.763	0.0008
**Round cell (M/mL)**	0.5667±0.0648	0.511±0.134	0.0784
**Morphologically normal spermatozoa (%)**	11.14±1.33	17.67±2.360	0.0498
**Teratozoospermia index**	1.561±0.034	1.484±0.055	0.2573
**Sperm deformity index**	1.841±0.062	1.703±0.105	0.1376
**Head deformity (%)**	79.64±2.109	70.29±4.079	0.0097
**Neck and midpiece deformity (%)**	46.99±2.086	39.65±2.585	0.0491
**Tail deformity (%)**	6.146±1.26	8.456±1.27	0.0431
**Excess residual cytoplasm (%)**	7.951±1.066	5.954±1.838	0.3044
**Normal acrosome (%)**	61.16±3.1	63.43±6.795	0.3915
**Abnormal acrosome (%)**	39.26±3.052	36.57±6.395	0.3648

* Values are represented as mean value ± Standard Error of the Mean (SEM)

*p*≤0.05 - statistically significant.

### HPV DNA detection in men from infertile and fertile couples

**Figure 2:**
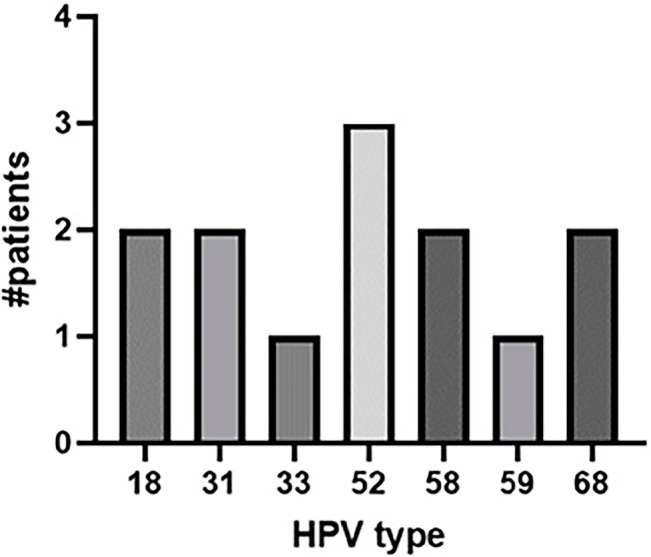
HPV typing results in men from infertile couples. Results are presented as the number of patients.

Seminal HPV DNA was detected in nine out of the 50 men from infertile couples (18.0%). The most prevalent type found was 52, detected in three subjects, accounting for 33.33% each. Types 18, 31, and 58 were present in 22.22% each, with two subjects affected by each. HPV types 33 and 59 were each identified in one subject. Among the potentially high-risk types, HPV 68 was the most prevalent, while no low-risk types were found. The HPV typing results, including high-risk and potentially high-risk types, are shown in Figure 2.

Out of the nine HPV-positive men from the infertile couples, six had single-type HPV detected (66.67%), and all of these were high-risk types (18, 31, 33, 58, 59). Two men (22.22%) had a mixed infection with two HPV types (52 - high-risk type, and 68 - potentially high-risk type), and one man (11.11%) had a concurrent presence of three high-risk HPV types (31, 52, 58).

In fertile couples, HPV was found in the semen of one out of ten men (10.0%), with a coinfection involving two types (HPV 16 and 31, both high-risk types).

### Microscopic semen parameters in HPV-positive (HPV+) and HPV-negative (HPV-) men from infertile couples

The results from the microscopic semen analysis in the HPV-positive and HPV-negative men from the infertile couples are shown in Table 2. The percentage of progressive motility was higher in HPV-positive men compared to HPV-negative men, and this difference was statistically significant (*p*=0.0399, t-test). The percentages of excess residual cytoplasm deformity, BCF, VSL, VAP, and LIN were all higher in HPV-positive men than in HPV-negative men from the infertile couples, and these differences were statistically significant (*p*=0.05, *p*=0.0239, *p*=0.0235, *p*=0.0434, *p*=0.05, t-test, appropriately). The percentage of sperm with normal acrosomes was higher in HPV-negative men (*p*=0.05, t-test), and WOB was higher in HPV-positive men compared to HPV-negative men (*p*=0.05, t-test).

**Table 2:** Microscopic semen parameters, HPV-positive vs. HPV-negative men from infertile couples.

Parameter	Mean value ± SEM HPV+ n=9	Mean value ± SEM HPV- n=41	*p*-value (t-test, Mann-Whitney)
**Sperm concentration (M/mL)**	30.68±7.001	30.07±6.397	0.1760
**Total sperm count (M/sample)**	70.15.43±15.18	71.02±11.81	0.2667
**Progressive motility (%)**	41.74±8.835	37.94±3.669	0.0399
**Total motility (%)**	53.89±8.457	53.52±3.905	0.4445
**Total motile sperm count (TMSC) (M/sample)**	35.80±11.98	33.47±6.937	0.2667
**VCL (µm/s)**	48.24±3.231	44.70±2.053	0.1841
**VSL (µm/s)**	24.56±1.826	20.51±0.9194	0.0235
**VAP (um/s)**	32.45±1.736	28.53±1.181	0.0434
**LIN (%)**	51.43±2.2557	46.78±1.923	0.05
**STR (%)**	71.52±1.607	66.87±2.142	0.073
**WOB (%)**	68.81±2.369	64.41±1.990	0.05
**ALH (µm)**	2.193±0.1524	2.135±0.097	0.4722
**BCF (Hz)**	6.973±0.2525	6.115±0.2273	0.0239
**Mucus penetration (%)**	20.93±5.369	15.29±1.759	0.2126
**Round cells (M/mL)**	0.5022±0.1321	0. 5780±0.07249	0.3587
**Morphologically normal spermatozoa (%)**	10.30±2.701	11.08±1.520	0.4875
**Teratozoospermia index (TZI)**	1.600±0.0602	1.553±0.04015	0.091
**Sperm deformity index (SDI)**	1.908±0.1536	1.827±0.0735	0.3826
**Head deformities (%)**	82.01±3.162	79.163±2.464	0.4641
**Neck and midpiece deformities (%)**	44.39±4.477	40.80±2.464	0.3207
**Tail deformities (%)**	5.293±1.585	6.321±1.467	0.4141
**Excess residual cytoplasm (%)**	10.19±2.168	7.492±1.203	0.05
**Normal acrosome (%)**	58.88±8.597	61.63±1.345	0.05
**Abnormal acrosome (%)**	41.12±8.597	38.87±3.286	0.40

* Values are represented as mean value ± Standard Error of the Mean (SEM), *p*≤0.05 - statistically significant.

### Microscopic semen parameters in HPV-positive men from infertile couples and men from fertile couples

**Figure 3:**
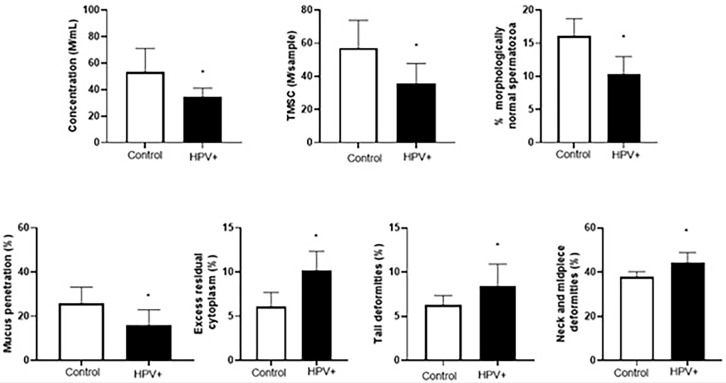
Comparison of microscopic semen parameters between HPV-positive men from infertile couples and men from fertile couples. The results are presented as mean ± SEM. *p≤0,05.

The results of the microscopic semen analysis in HPV-positive men from infertile couples and men from fertile couples are shown in Figure 3. A higher sperm concentration was observed in the fertile controls compared to HPV-positive men from infertile couples, and this difference was statistically significant (*p*=0.0493, t-test). Both TMSC and mucus penetration were lower in HPV-positive men from infertile couples compared to men from fertile couples, and these differences were statistically significant (*p*=0.0291 and *p*=0.0088, respectively, t-test). Additionally, statistically significant differences were observed in morphology, with the percentage of morphologically normal sperm being lower in HPV-positive men from infertile couples compared to men from fertile controls (*p*=0.05, t-test). The percentages of excess residual cytoplasm (*p*=0.05, t-test), tail deformities (*p*=0.05, t-test), and neck and midpiece deformities (*p*=0.0499, t-test) were all significantly higher in HPV-positive men from infertile couples compared to men from fertile controls.

## DISCUSSION

HPV infections are the most common viral sexually transmitted infections. Men serve as a significant reservoir for the virus. Globally, one in three men is infected with at least one HPV genotype (estimated global prevalence: 31%), with genotype 16 being the most frequently detected, followed by types 6, 51, 52, 59, and 18 (Bruni *et al*., 2023). HPV DNA has been detected at various anogenital sites in men. In the context of male infertility, HPV DNA has been found in all ejaculate fractions, and virions have been localized at the equatorial segment and midpiece of spermatozoa (Capra *et al*., 2019; Kato *et al*., 2021; Notari *et al*., 2024; Giovannelli *et al*., 2007; Kaspersen *et al*., 2011; Foresta *et al*., 2010). The lifetime probability of men acquiring an HPV infection is estimated to be as high as 90% (Garolla *et al*., 2023).

In our study, the prevalence of seminal HPV infection among infertile men was 18.0%, which aligns with previous reports (Weinberg *et al*., 2020). One man from the fertile group had a mixed infection with three HPV genotypes, a finding consistent with previous studies involving European sperm donors co-infected with multiple HPV types (Depuydt *et al*., 2019; Kaspersen *et al*., 2011). This individual also presented with genital warts, and seminal HPV prevalence among such men may reach up to 54% (Foresta *et al*., 2010).

Kinematic parameters-including VCL, VSL, VAP, BCF, and ALH-were significantly lower in infertile men compared to fertile controls. Although standardized reference values for these parameters remain under discussion, high values of VSL, VCL, and ALH are usually linked to activated or hyperactivated spermatozoa. ALH, in particular, indicates the force of flagellar beating along with the rotation frequency of the spermatozoa, which correlates with their ability to penetrate cervical mucus and fuse with the oocyte (Mortimer & Mortimer, 2013; Fernández-López *et al*., 2022, Aghazarian *et al*., 2021). As expected, mucus penetration ability was also reduced in the infertile group. Furthermore, infertile men showed a significantly lower percentage of morphologically normal spermatozoa, along with increased rates of tail and neck/midpiece abnormalities.

Interestingly, among infertile men, those with HPV-positive semen showed significantly higher progressive motility than HPV-negative men. This finding contradicts previous reports, which generally associate HPV infection with reduced sperm motility (Weinberg *et al*., 2020; Moreno-Sepulveda & Rajmil, 2021; Das *et al*., 2023; Boeri *et al*., 2019; Cao *et al*., 2020). One possible explanation is that lower BCF values in HPV-negative men may indicate more efficient motility due to stronger flagellar force and less head oscillation, leading to higher progressive motility (Fernández-López *et al*., 2022). However, in our study, the HPV-negative group showed lower progressive motility. It is important to note that total progressive motility was assessed without distinguishing between rapid and slow components. Notari *et al*. (2024) previously reported that HPV-positive semen might display increased rapid progressive motility, potentially linked to enhanced mitochondrial metabolic activity, which could also explain our findings.

Additionally, in the infertile group, HPV-positive men showed higher VSL, VAP, LIN, and WOB compared to HPV-negative men. Since linearity is one of the parameters used to characterize capacitated and hyperactivated sperm movement (Van der Horst & Du Plessis, 2017), these findings may indicate that HPV makes sperm more resistant to capacitation and hyperactivation. One potential mechanism by which HPV could influence the likelihood of achieving spontaneous pregnancy and the success of intrauterine insemination is through increasing sperm movement linearity, which may impair hyperactivation. Sperm that move in a linear rather than a hyperactivated, ‘big star-like’ pattern may become trapped in the crypts and folds of the endometrial and oviductal epithelium, ultimately failing to reach the oocyte.

The significantly lower sperm concentration seen in HPV-positive men from infertile couples compared to fertile controls aligns with previous research and may be due to HPV-driven inhibition of Aquaporin-8, which controls sperm volume and oxidative stress regulation. This could make sperm more prone to oxidative damage, leading to a reduced sperm count (Pellavio *et al*., 2020; Wang *et al*., 2021; Weinberg *et al*., 2020).

Additionally, total motile sperm count (TMSC) and mucus penetration were significantly lower in HPV-positive men compared to fertile controls. TMSC is widely recognized as a reliable indicator of the severity of male infertility and as a predictor of success in both natural and assisted conception (Pellavio *et al*., 2020; Hamilton *et al*., 2015). Reduced mucus penetration may result from HPV-induced suppression of sperm hyperactivation.

Morphologically, HPV-positive men from infertile couples showed significantly lower proportions of normal sperm and higher rates of abnormalities-especially excess residual cytoplasm, tail deformities, and neck/midpiece defects-compared to fertile men. These findings align with prior meta-analyses indicating that HPV infection adversely affects sperm morphology and motility (Wang *et al*., 2021; Weinberg *et al*., 2020; Boeri *et al*., 2019). The increase in tail and neck deformities may also reduce overall sperm motility. Furthermore, HPV-induced inhibition of Aquaporin-8 results in dysregulation of sperm volume and hampers the removal of excess residual cytoplasm during spermatogenesis, both of which can impact sperm morphology.

A primary limitation of this study is the relatively small sample size, especially within the proven-fertility control group, which consisted of healthy individuals visiting the clinic for non-infertility-related concerns. This could explain their low interest in participating in the study. Additionally, total progressive motility was measured as a single parameter; differentiating between rapid and slow progressive motility could have provided more detailed insights into the observed patterns. Moreover, lifestyle factors were noted but not controlled for in the study. Nonetheless, this study used advanced semen testing methods (CASA), and the impact of HPV on kinematic parameters was examined. The subgroup analyses comparing HPV-positive and HPV-negative men within the infertile group allow for more precise attribution of findings to HPV presence, especially with the inclusion of fertile controls in the study design.

## CONCLUSION

In conclusion, the presence of HPV in the ejaculate of men from infertile couples significantly impacts sperm function, as evidenced by changes in key microscopic parameters - sperm concentration, motility, and morphology. This supports considering HPV as a potential factor in male infertility. HPV screening could be added to the diagnostic process for infertile men and may serve as a new therapeutic target.
